# The Soft Palate Enables Extreme Feeding and Explosive Breathing in the Fin Whale (*Balaenoptera physalus*)

**DOI:** 10.1093/iob/obae026

**Published:** 2024-07-09

**Authors:** A W Vogl, H Petersen, K N Gil, R L Cieri, R E Shadwick

**Affiliations:** Life Sciences Centre and Department of Cellular and Physiological Sciences, University of British Columbia, Vancouver, BC V6T 1Z3, Canada; Department of Anatomy, Medical Faculty, University of Iceland, 101 Reykjavik, Iceland; Department of Surgery, Akureyri Hospital, 600 Akureyri, Iceland; Department of Zoology, University of British Columbia, Vancouver, BC V6T 1Z4, Canada; Department of Zoology, University of British Columbia, Vancouver, BC V6T 1Z4, Canada; Department of Zoology, University of British Columbia, Vancouver, BC V6T 1Z4, Canada

## Abstract

The evolution of lunge feeding in rorqual whales was associated with the evolution of several unique morphological features that include non-synovial ligamentous temporomandibular joints, a tongue that can invert and extend backward to the umbilicus, walls of the oral cavity that can dramatically expand, and muscles and nerves that are stretchy. Also, among the acquired features was an enlargement of the rostral end of the soft palate into an oral plug that occludes the opening between the oral cavity and pharynx and prevents water incursion into the pharynx during the engulfment phase of a feeding lunge. During this engulfment phase of a lunge, the volume of water entering the oral cavity can exceed the volume of the whale itself. Here, using dissection of fetuses and adults and a magnetic resonance imaging dataset of a fetus, we examine the detailed anatomy of the soft palate in fin whales. We describe several innovative features relative to other mammals, including changes in the attachment and positions of the major extrinsic muscles of the palate, alterations in the morphology of the pterygoid processes related to the palate and pharynx, and the presence of distinct muscle layers in the part of the palate caudal to the oral plug. Based on the anatomy, we present a model for how the soft palate is positioned at rest, and how it functions during feeding, breathing, and swallowing.

## (Icelandic abstract) Samantekt

Þróun gleypi-kynginga við fæðuöflun reyðarhvala hefur haft í för með sér breytingar á mörgum einstökum byggingarþáttum svo sem kjálkaliðs án hálahimnu, tungu sem leyfir viðsnúning svo henni má kyngja, en þá nær tungubroddurinn niður undir nafla, veggi munnhols sem hafa gríðarlega þangetu og Þar með teygingu vöðva, æða og tauga. Til viðbótar við þessa þróun var stækkun á dausenda mjúkagóms í nokkurs konar kverkartappa (e. oral plug) sem myndar lokun milli munnhols og koks sem hindrar að vatn (sjór) sleppi niður í kok í gleypifasa fæðunáms, sem felur í sér hám á miklu magni í einu. þetta magn sem gleypt er, er sjór sem í er blönduð sú bráð sem sóst er eftir hverju sinni, en magn sjávarins að þyngd getur jafnast á við þyngd þess hvals sem veiðir. Í þessari grein var beitt krufningum og segulóm- og tölvusneiða myndgreiningu til að rannsaka nákvæmlega líffærafræðilega byggingu mjúkagóms og koks skíðishvala. Lýst er hér nokkrum, áður óbirtum og ólíkum byggingarþáttum, samanborið við önnur spendýr, svo sem stöðu og festu stærstu vöðva mjúkagóms, útlitsbreytingum er taka til vængklakks og áberandi vöðvalags dauslægt við kverktappann. Byggt á þessari líffærafræðilegu byggingu er kynnt model sem skýrir stöðu og starf mjúkagóms í hvíld, við öndun og við gleypi-kyngingu.

## Introduction

**Fig. 1:**
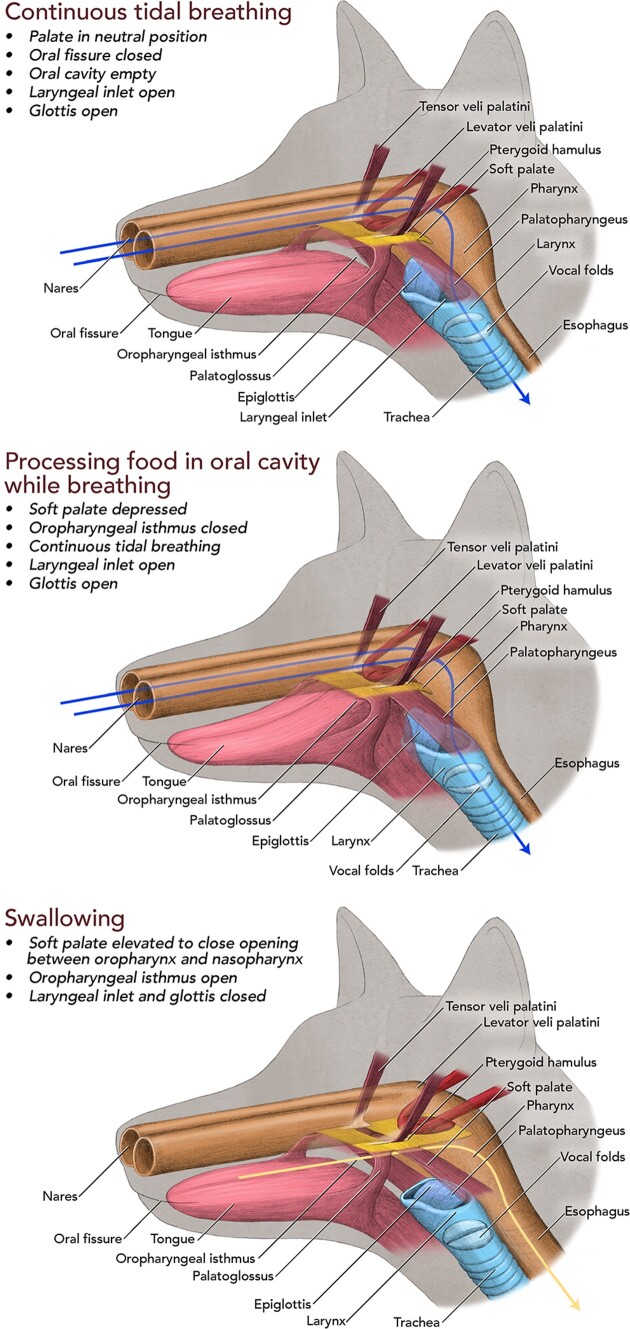
The general structure and function of the mammalian soft palate while tidal breathing, while processing food or liquid in the oral cavity, while breathing, and while swallowing. The lines in the upper and middle panels indicate the air pathway when breathing. The line in the lower image indicates the food pathway when swallowing. Artwork © 2024 Alex Boersma (used with permission).

The transition from an aquatic to a terrestrial habitat around 400 million years ago had a profound influence on vertebrate morphology. Among the numerous adaptations that evolved were an air-based respiratory system that developed as an outgrowth of the “gut tube,” and a series of valve mechanisms to functionally separate the airway from the food pathway. Based on the anatomy of existing fish that have respiratory swim bladders or “lungs” (Cupello et al. 2017; Scadeng et al. 2020; Cupello et al. 2022), the first valve to evolve was likely a muscular sphincter at the point where the airway branches from the gut tube. As the airway was reinforced with a cartilage, the muscular sphincter was replaced with a primitive larynx, consisting of two arytenoid cartilages that can be moved, by dilator and constrictor muscles, on an adjacent cricoid (cricoid-like) cartilage to open and close the inlet (glottis) to the lower airway. This is generally the situation in most existing amphibians, birds, and non-avian reptiles (Trewavas 1933; Hilton 1952; Young 2000; Homberger 2017; Russell and Bauer 2021). Later, in mammals, a thyroid cartilage and epiglottis evolved as part of the laryngeal structure, and a soft palate evolved as an extension of the newly acquired hard palate (Parrington and Westoll 1940; Smith 1992). Depression of the soft palate closes the opening (oropharyngeal isthmus) between the oral cavity and pharynx to allow breathing while processing food in the oral cavity (Fig. 1). Elevation opens the oropharyngeal isthmus allowing food/liquid to move through the pharynx and into the esophagus while at the same time closing the opening (pharyngeal isthmus) between the nasopharynx and oropharynx preventing material from moving into the upper airway (nasopharynx and nasal cavities). Movement of the epiglottis to close the superior opening of the larynx supplements closure of the glottis (*rima glottidis*) by the arytenoids and associated soft tissues during swallowing (Fig. 1).

Four major extrinsic muscles on each side contribute to the structure and function of the soft palate in a typical mammal (Fig. 1). On each side, a tensor muscle (*M tensor veli palatini*) originates mainly from the sphenoid bone at the base of the skull and then descends ventrally to form a tendon that loops 90° around the hamulus of the pterygoid process (*hamulus pterygoideus*) of the sphenoid bone. The tendon courses medially, expands, and joins with its partner from the other side to form an aponeurotic platform to which the three other muscles of the soft palate attach. A levator muscle (*M levator veli palatini*) originates from the base of the skull caudal to the tensor and mainly from the temporal bone. This muscle descends to insert directly into the soft palate and functions in elevation. Two other muscles, palatoglossus (*M palatoglossus*) and palatopharyngeus (*M palatopharyngeus*), on each side course ventrally from the soft palate and insert one into the lateral side of the tongue, and the other into the lateral aspect of the pharyngeal wall, respectively. These muscles depress the palate, and together with elevation of the back of the tongue, function to close the oropharyngeal isthmus.

The movement back into a totally aquatic environment by the Cetacea around 50 million years ago also had a dramatic influence on body form, including modifications to the valving systems that work to separate the respiratory system from the digestive tract. Unlike in terrestrial mammals that are continuous tidal breathers (i.e., continuous inhalation and exhalation at a constant rate and volume), and the airway is generally always open except during swallowing, the Cetacea are periodic explosive breathers, and the default position of valving systems in the airway is closed except when breathing. In toothed whales (odontocetes) the respiratory tract is completely separated from the digestive tract. In this group, the cranial end of the larynx is elongated and telescopes into the nasopharynx where it is held into position by a muscular sphincter formed by the *palatopharyngeus* muscles (Reidenberg and Laitman 1987). In these animals, food items pass through the pharynx on either side of the larynx and into the esophagus. Something quite different occurs in the rorqual group (Balaenopteridae) of baleen whales, most likely related to their novel feeding strategy.

The rorqual group of whales includes two of the largest animals that have ever lived (blue whales [*Balaenoptera musculus*] and fin whales [*Balaenoptera physalus*]). Unlike other baleen whales, rorqual whales are characterized by “pleated grooves” (ventral grooved blubber) present on the ventral aspect of their bodies that extend posteriorly from the chin to as far back as the umbilicus and that allow expansion of the oral cavity during feeding. Rorquals are lunge feeders and engulf huge quantities of prey-laden water during each lunge; in fact, the volume of water engulfed can be larger than the volume of the whale itself (Goldbogen et al. 2007, 2010). During a lunge, the tongue inverts and expands backward into a ventral fascial pouch that extends as far caudally as the umbilicus. During a single feeding dive, a whale can make multiple lunges with the interval between the initiation of each successive lunge being less than a minute (Goldbogen et al. 2006)—a time that includes engulfment, filtration, and swallowing. When the mouth (oral fissure; *rima oris*) closes after an engulfment, muscles associated with the ventral grooved blubber and floor of the oral cavity contract and concentrate prey by expelling water through baleen plates attached to the upper jaw. The concentrated prey then moves from the oral cavity into the pharynx and through a small-diameter esophagus to the stomach. Rorquals face three major issues during feeding: (1) preventing prey-laden water from passing from the oral cavity into the pharynx during engulfment and filtration; (2) swallowing a large volume of concentrated prey in a short time between initiating lunges; and (3) preventing food from entering the lower airway when swallowing. Among the anatomical features critically important to solving these issues is the soft palate.

Little is known about the anatomy of the soft palate in rorquals; however, one significant feature that is known is the presence of a dramatic thickening or enlargement of the rostral end into an “oral plug” that completely occludes the opening between the oral cavity and pharynx (oropharyngeal isthmus) (Gil et al. 2022; Lauridsen et al. 2022). Based on dissections of adult animals and fetuses, and a magnetic resonance imaging (MRI) scan of a fetus, we explore the general features and major muscles of the soft palate in the fin whale (*B. physalus*) and present a model for how it may function to facilitate engulfment/filtration, swallowing, and breathing.

## Methods

The soft palate and related structures of two adult fin whales (female, length 19.26 m; male, length 17.68 m) were dissected in detail during the summer of 2023 at the commercial catch station at Hvalfjörður, Iceland. In addition, the relevant anatomy of several other adult individuals was examined and photographed in less detail during the summers of 2014 and 2023 (females, lengths 18.55, 17.82, 19.16, 18.7, and 19.2 m; males, lengths 17.44, 18.87, 17.00, and 18.38 m). During the fall of 2021, two archived fetuses (female, length 201 cm; male, length 198 cm) were dissected at the station after an imaging dataset of the head and neck of the larger fetus was collected by MRI. The fetuses were frozen when initially collected and were thawed prior to imaging and dissection.

The MRI dataset was obtained using a Siemens 3T Magnetom Prisma. The dataset was analyzed using OsiriX MD software. A 3D image of the soft palate was created by manually outlining with the pencil tool the structure as a “region of interest” in selected sequential images and then using the software to create the final 3D rendering. The image was rotated to obtain optimal views of the different regions of the developing palate, and the final images were captured as screenshots.

The caudal region of the base of a fin whale skull in pristine condition was examined and photographed. The bones are from a juvenile male (length 12.8 m) that had washed up on the coast of British Columbia and are now in the collection of the Irvin Louis Museum of the shíshálh Nation.

## Results

### Fetuses (MRI and dissection)

**Fig. 2:**
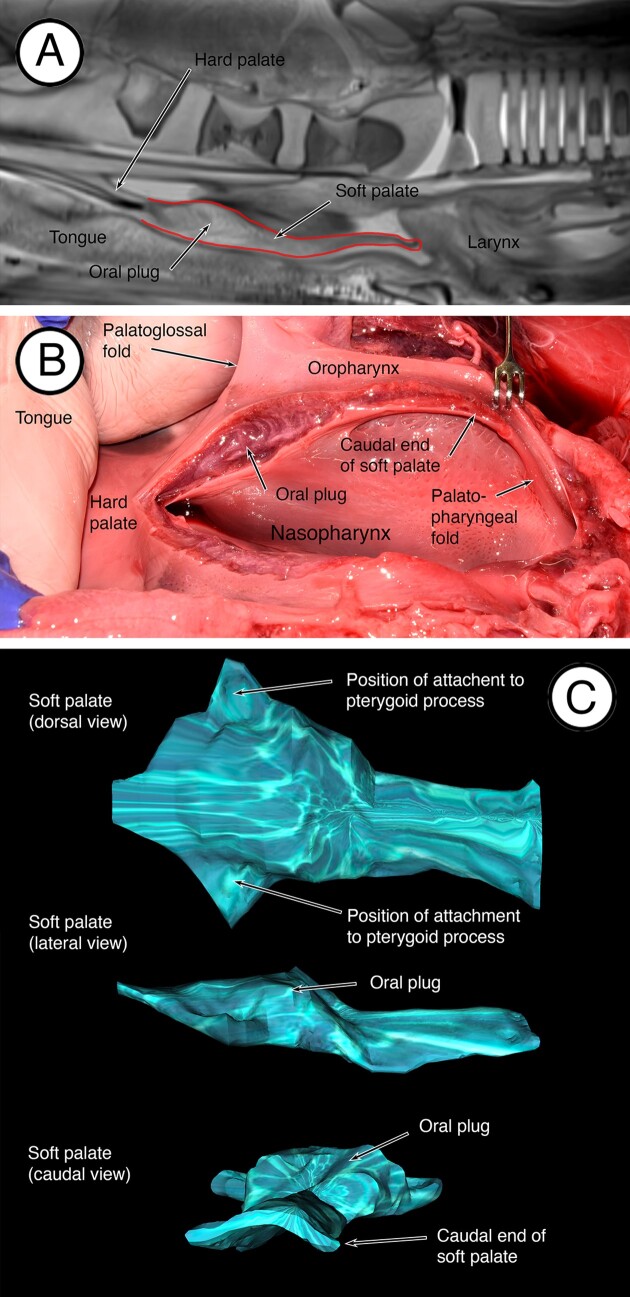
Developing soft palate in fetal fin whales. (**A**) MRI scan in the mid-median plane showing the soft palate (outlined) with the developing oral plug at the rostral end. The rostral end of the larynx is positioned just above the caudal free edge of the soft palate. (**B**) In this dissection, the fetus is on its back. The soft palate has been sectioned in the mid-median to show the developing oral plug at the rostral end. The palatopharyngeal fold merging with the pharyngeal wall is evident at the caudal end of the soft palate. (**C**) Reconstructions of the soft palate created from the MRI dataset using OsiriX MD software.

In fetuses, the soft palate and related structures are evident in the MRI scans (Fig. 2A) and in dissections (Fig. 2B). In MRI images in the median plane (Fig. 2A), the soft palate is anchored to the hard palate rostrally and extended caudally in the horizontal plane to terminate as a free margin in close association with the larynx. The cranial end of the larynx is slightly above the caudal margin of the soft palate. In dissections, the caudal margin of the soft palate is anchored to the pharyngeal wall by palatopharyngeal folds, and rostrally is attached to the tongue by palatoglossal folds (Fig. 2B). The developing oral plug is evident as an enlargement or thickening of the rostral end of the soft palate. When the soft palate is reconstructed in 3D from the MRI scans using OsiriX MD software, the oral plug is evident as a dorsal enlargement of the soft palate and is anchored to the pterygoid processes (*processus pterygoideus*) of the sphenoid bone laterally on each side (Fig. 2C). Caudal to the developing oral plug, the soft palate tapers to a flatter sheet.

**Fig. 3:**
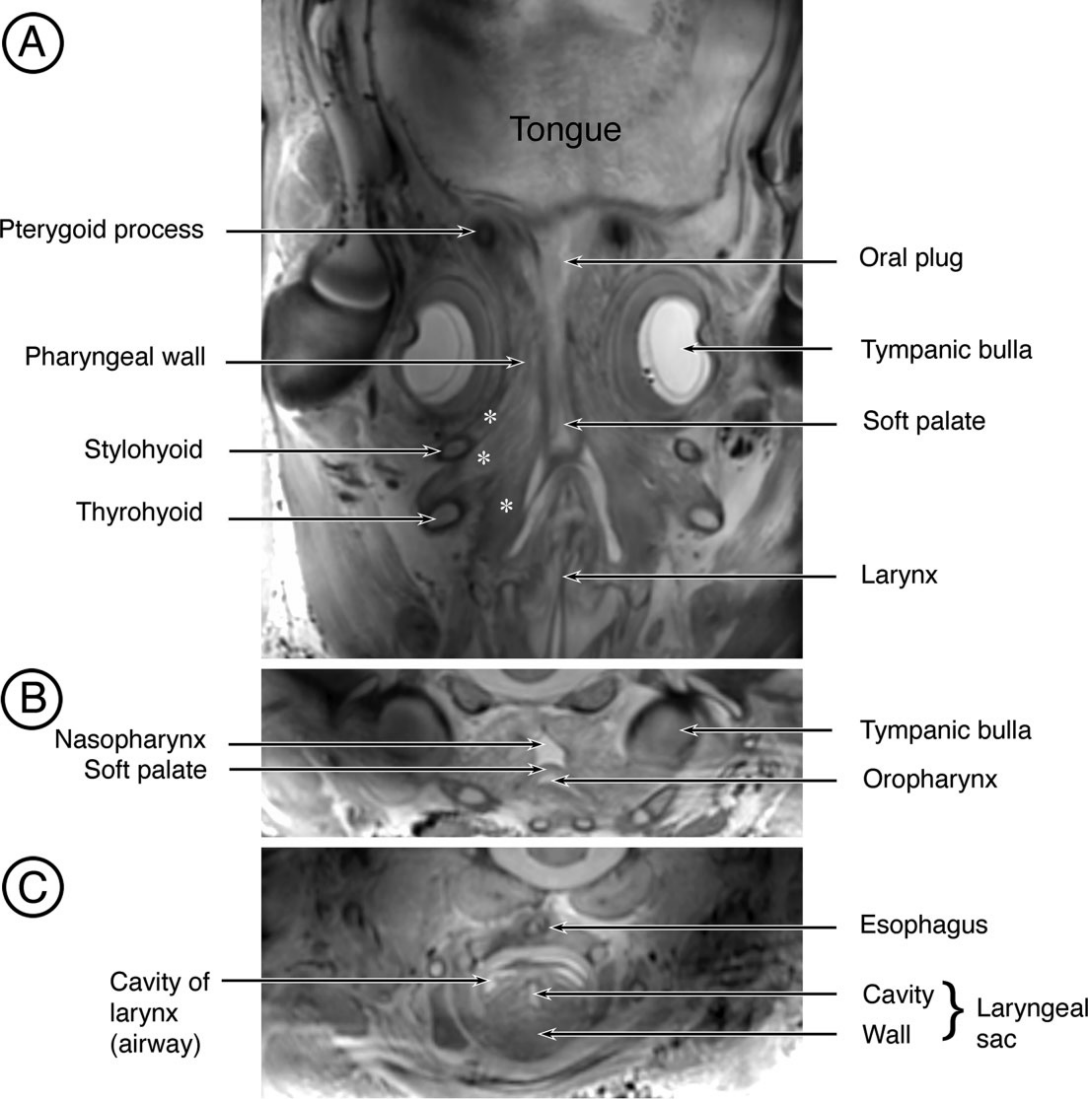
MRI scans of the soft palate and pharynx in a fetal fin whale. (**A**) MRI scan in the dorsal plane through the pharynx and soft palate showing the relationship of the soft palate and pharyngeal wall to the pterygoid processes and larynx. Muscles (middle and inferior constrictors) of the pharyngeal wall attaching to the hyoid apparatus and larynx are indicated by the asterisks. (**B**) Transverse section through the oropharynx, nasopharynx, and soft palate at a level near the caudal end of the tympanic bullae. (**C**) Transverse section through the larynx below and the beginning of the esophagus above. Also visible in this image is the laryngeal sac.

**Fig. 4:**
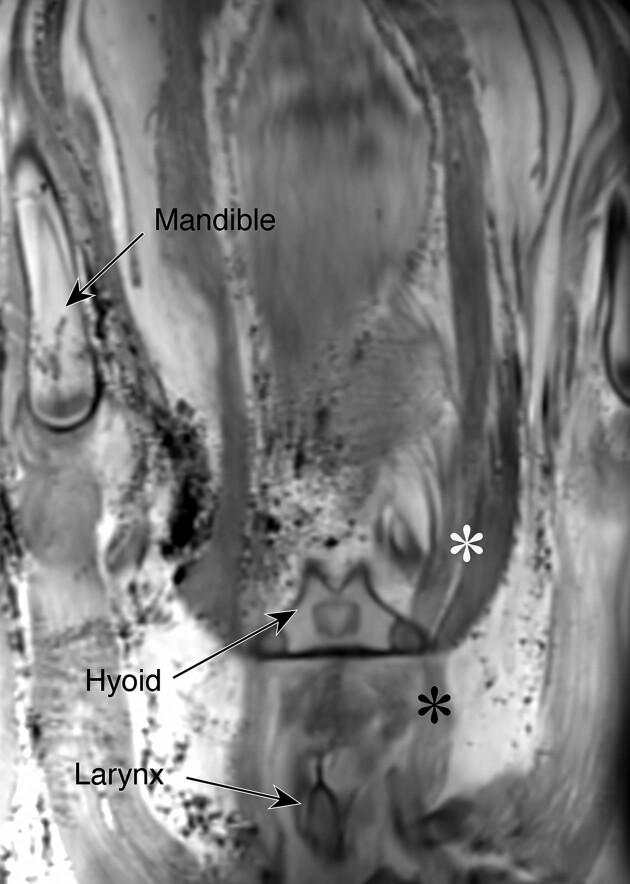
MRI scan in the dorsal plane to illustrate muscles associated with the hyoid apparatus in a fetal fin whale. Suprahyoid muscles are indicated by the white asterisk, and infrahyoid muscles are indicated by the black asterisk.

In sections in the dorsal plane from the MRI scans (Fig. 3A), the attachments of the soft palate anteriorly to the pterygoid processes and laterally to the pharyngeal wall are evident, as is the relationship caudally to the larynx. Also evident are large middle and inferior constrictor muscles of the pharyngeal wall that attach to the hyoid apparatus and larynx, respectively (asterisks in Fig. 3A). When the scans are viewed in the transverse plane in the region of the tympanic bullae, the shelf-like soft palate separates the nasopharynx above from the oropharynx below (Fig. 3B), and in sections further caudally through the larynx, the small esophagus is evident in a dorsal position to the airway (Fig. 3C). Also evident in the latter is the prominent laryngeal sac. In a section in the dorsal plane through the hyoid, prominent suprahyoid and infrahyoid muscles are visible (Fig. 4).

**Fig. 5:**
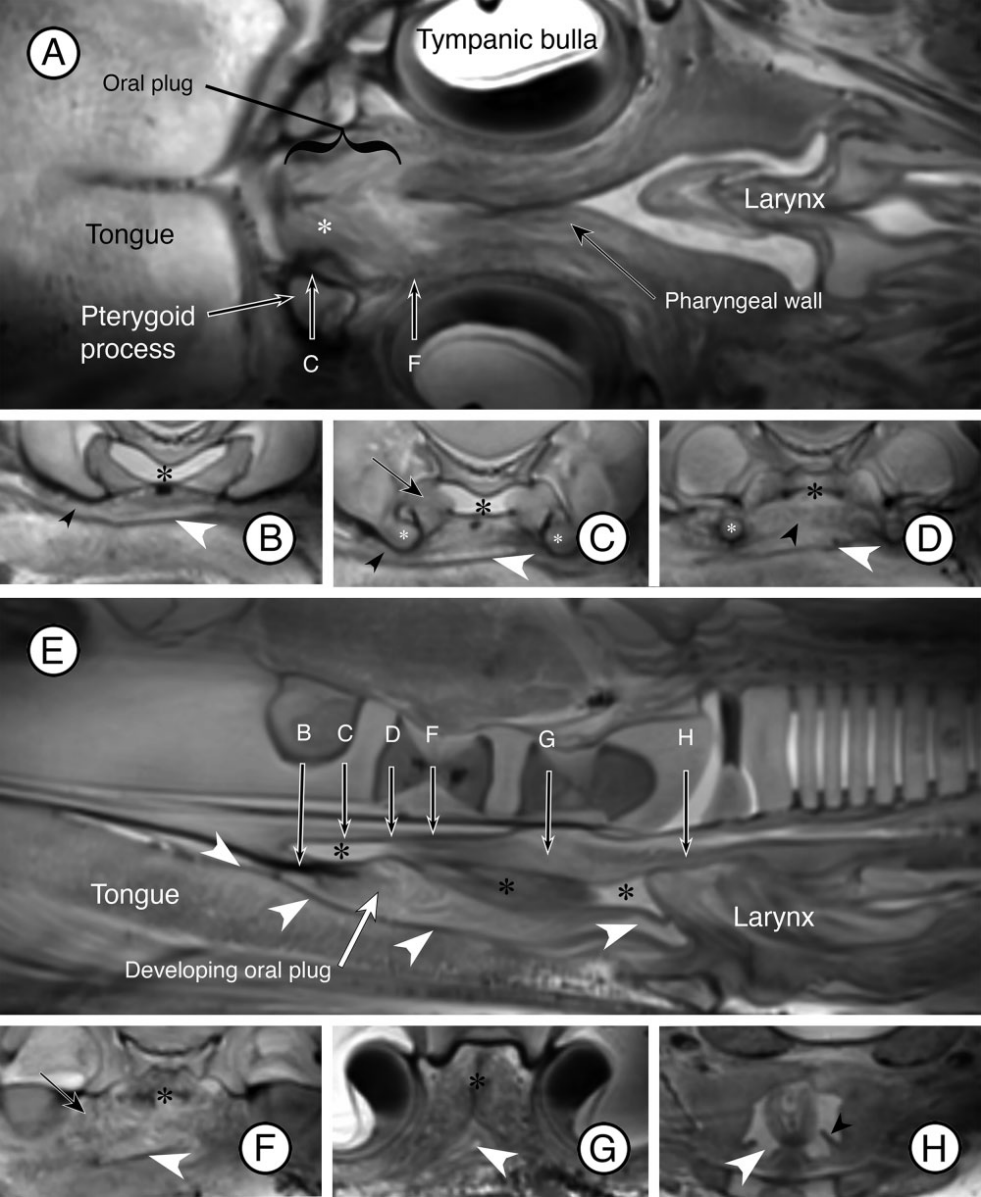
MRI scans that show developing muscles and mucosal folds of the soft palate in a fetal fin whale. Images in the dorsal and mid-median planes are shown in (**A**) and (**E**), respectively. Arrows with letters in these images are the approximate positions at which the corresponding images in the transverse plane (**B–D; F–H**) are taken. Dorsal is up in these transverse sections, and the black asterisks indicate the airway, and the white arrowheads indicate the “food” or “gut” pathway. In (**B, C**) the small black arrowheads indicate the developing palatoglossal fold and muscle. In (**C, D**) the small white asterisks indicate the developing pterygoid processes. In (**C**) the part of the developing levator veli palatini is indicated by the arrow, which is also visible near the asterisk in (**A**). In (**D**) the small black arrowhead indicates the developing tensor veli palatini muscle between the two pterygoid processes. In (**F**), the arrow points to another part of the developing levator veli palatini muscle, which is also indicated by the white asterisk in (**A**). In (**H**) the small black arrowhead points to one of the palatopharyngeal folds.

Some of the more detailed anatomical features of the soft palate are revealed on closer inspection of the MRI scans when viewed in dorsal (Fig. 5A) and mid-median (Fig. 5E) planes, and in related transverse sections (Fig. 5B–D, F–H). In dorsal sections (Fig. 5A), the pterygoid processes of the sphenoid are evident lateral to the oral plug and give attachment to both the soft palate and the pharyngeal wall. On each side, a developing muscle is attached to bone both at and just caudal to the pterygoid process (marked by the asterisk in Fig. 5A) and descends into the oral plug when seen in the transverse section (arrows in Fig. 5C, F). This muscle would elevate the oral plug and is a candidate for the levator veli palatini muscle. Tissue extending anterolaterally on each side into the tongue is likely the developing palatoglossal muscle within the palatoglossal fold (small black arrowheads in Fig. 5B, C). Evident within the oral plug and extending medially or horizontally from the end of the pterygoid process on each side is tissue that likely represents the tensor veli palatini muscle (small black arrowhead in Fig. 5D). This developing muscle lacks a vertical component descending from the base of the skull and attaches directly to the ventral end of the pterygoid process. The caudal end of the soft palate is anchored on each side to the pharyngeal wall by a small palatopharyngeal fold (small black arrowhead in Fig. 5H). In the mid-median section (Fig. 5E) and in transverse sections (Fig. 5B–D, F, G), the airway (marked by the black asterisks) and “food” pathway (marked by the white arrowheads) are visible.

### Adult

**Fig. 6:**
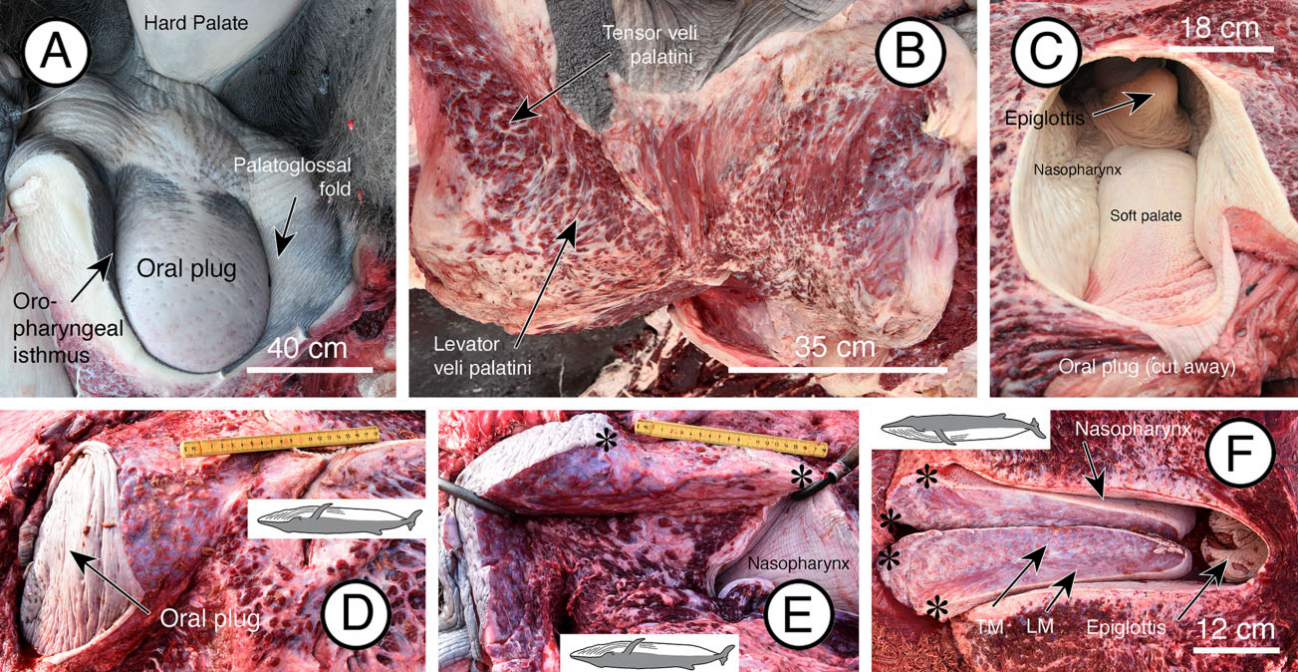
Images of the soft palate in adult fin whales. (**A**) View from the opened oral cavity of the oral plug at the rostral end of the soft palate. The plug completely fills the oropharyngeal isthmus, which is visible only as a slit around the ventral and lateral margins of the plug. (**B**) Similar view as in (**A**), but with the plug sectioned in the median plane with the two sides pulled apart so that the internal structure is visible. The tensor veli palatini muscle is indicated by the white asterisk and the levator veli palatini indicated by the black asterisk. (**C**) Dorsal view, from the nasopharynx, of the soft palate caudal to the oral plug that has been cut away. (**D**) Lateral view of the oral plug (dorsal is down in the image). (**E**) Mid-median section through the oral plug (dorsal is down in the image). Asterisks indicate where the caudal part of the soft palate has been cut away from the oral plug. (**F**) Mid-median section through the soft palate caudal to the oral plug and illustrating the constituent dorsal longitudinal (LM) and ventral transversal (TM) arrangement of skeletal muscle layers (dorsal is up in the image). The palate is viewed from a dorsal position. Asterisks indicate where the soft palate has been cut away from the oral plug.

In adult fin whales, the oropharyngeal isthmus is completely closed by the oral plug except when swallowing (Fig. 6A). The plug is composed of muscle and fat when sectioned in the median plane (Fig. 6B), and the soft palate extends caudally as a thin shelf from the plug to the epiglottis of the larynx as in the fetus (Fig. 6C). When further dissected and sectioned in the median plane (Fig. 6A–C), the soft palate caudal to the plug is thin, consists of muscle (dorsal longitudinal and ventral transverse layers) and adipose tissue, and tapers to end as a free edge just anterior to the larynx where the palate is attached to the pharyngeal wall by thin palatopharyngeal folds (see fig. 2B in Gil et al. 2020).

**Fig. 7:**
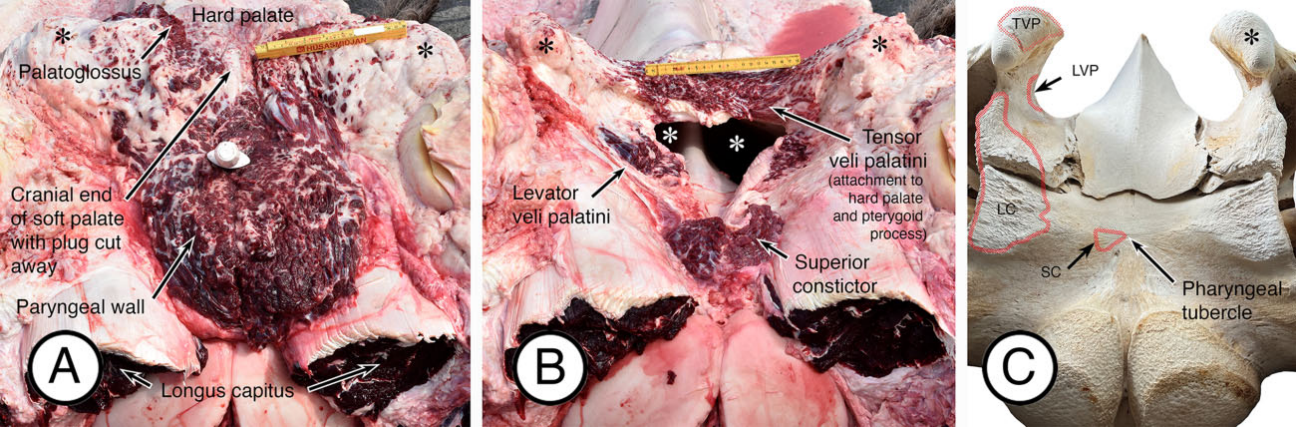
Muscles of the soft palate and of associated regions in the adult fin whale (ventral view). (**A**) Position of the cranial attachment of the soft palate, with most of the oral plug removed, and of the cranial end of the pharyngeal wall. The white handle is visible of a dissection tool inserted into the cranial end of the nasopharynx. The pterygoid processes are indicated by the black asterisks (in all three panels), and the attachment of palatoglossus muscle to the palate is labelled. The large longus capitis muscles are also labeled. (**B**) Further dissection of the material shown in (**A**). The attachments of the most cranial parts of tensor veli palatini muscles to the hard palate are indicated by an arrow and labelled. One of the levator veli palatini muscles is also indicated by an arrow and labelled, as is the superior constrictor muscle of the pharynx. The choanae are indicated by the two white asterisks. (**C**) Ventral view of the posterior aspect of a juvenile fin whale skull with the bony attachments of the muscles shown in (**B**) indicated. TVP—tensor veli palatini; LVP—levator veli palatini; LC—longus capitis; SC—superior constrictor; asterisk—pterygoid process.

In detailed dissections of the adult oral plug and related regions (Fig. 7A, B), candidates for the palatoglossus, tensor veli palatini, and levator veli palatini muscles were identified. As observed in the fetus, the tensor muscles lack a vertical component and the horizontal part attaches directly to the ventral end of the pterygoid process and to the hard palate, and then descends into the plug (see Fig. 6B). The levator muscles descend into the plug from an attachment on the cranial aspect of the pterygoid process where the process originates from the base of the skull. Particularly noticeable in these dissections was the thickness of the pharyngeal wall in the region of the plug, and the attachment of the large longus capitis muscle on each side just lateral to the pharyngeal wall. Also observed was the attachment of the superior constrictor of the pharynx on each side just lateral to the pharyngeal tubercle of the occipital bone.

In an isolated bone preparation consisting of the occipital, sphenoid, and vomer from a juvenile male fin whale (Fig. 7C), the pterygoid processes are present as robust ventral peg-like extensions of the sphenoid bone that are positioned just posterior and lateral to the choanae. The ventral ends of each process extend caudally as substantial prong-like structures that provide attachment both for the pharyngeal wall and for the oral plug of the soft palate. This is unlike the situation in odontocetes where the ventral ends of the pterygoid processes course medially as palatine plates and fuse at the midline as part of the hard palate (Mead and Fordyce 2009).

## Discussion

### Anatomical findings

The soft palate of the fin whale is part of the constellation of anatomical adaptations that facilitate a lunge feeding strategy that is unique to rorquals. One major innovation to the soft palate is an enlargement of the cranial end of the soft palate to form an oral plug that occludes the oropharyngeal isthmus except during swallowing. The four major extrinsic muscles (tensor veli palatini, levator veli palatini, palatoglossus, and palatopharyngeus) that generally occur in the soft palate of mammals are present in the fin whale, but have some features associated with the presence of the plug and the functional requirements of having to elevate the plug to open the oropharyngeal isthmus during swallowing. One related adaptive feature is the robust structure of the pterygoid processes of the sphenoid bone that provides support for the oral plug and for the attachment of the thick muscular lateral pharyngeal wall (superior constrictor).

Generally, in mammals, the pterygoid processes of the sphenoid bone project inferiorly as vertical plates positioned lateral to and bracketing the caudal openings (choanae) of the nasal cavities. They often provide significant attachments for parts of the pterygoid muscles that, among other functions, participate in generating rotational chewing movements of the mandible. The processes also provide cranial attachment for the pharyngeal wall to the lateral bony margins of the nasal cavities. At their ventral caudal ends, each pterygoid process has a hook-like projection or hamulus around which the tendon of tensor veli palatini muscle curves to course medially, often as aponeurosis, into the soft palate.

In the fin whale, the pterygoid processes are substantial peg-like structures and lack any form of hamulus. Also, and as reported in another rorqual whale (sei whale—*Balaenoptera borealis*) (Schulte 1916), the pterygoid muscles have no substantial origin from the pterygoid processes. As a result, the pterygoid processes are related solely to supporting the oral plug of the soft palate and the thick muscular wall of the pharynx.

**Fig. 8:**
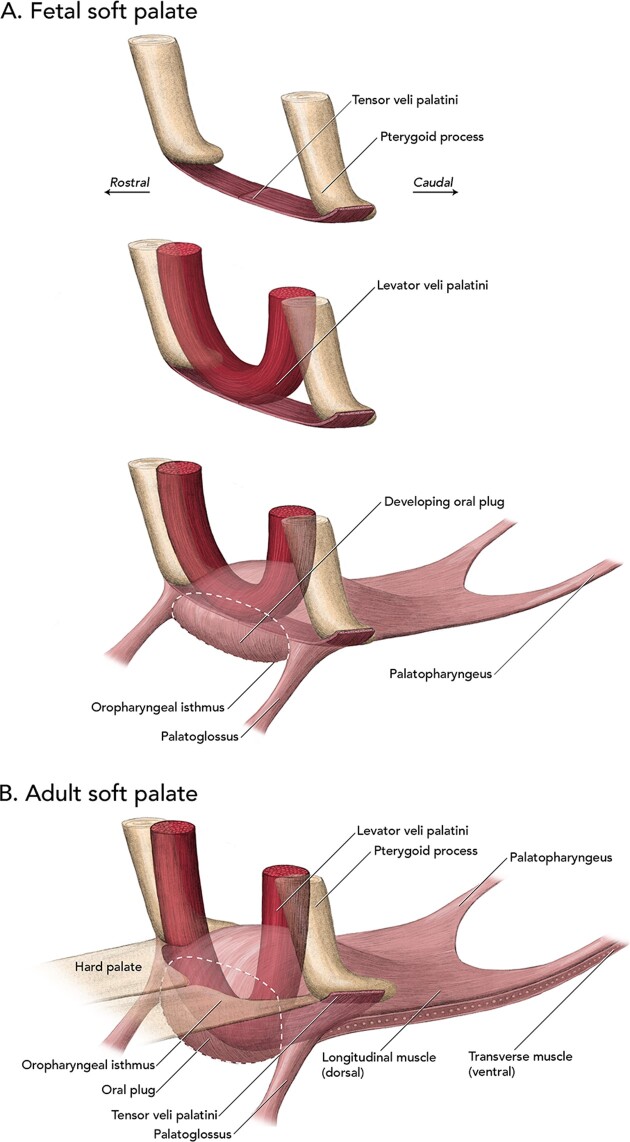
Summary of muscles and associated structures in the soft palate of fetal (**A**) and adult (**B**) fin whales. Artwork © 2024 Alex Boersma (used with permission).

Both the tensor veli palatini and levator veli palatini muscles originate in relationship to the pterygoid processes in the fin whale. In the fetus, each tensor veli palatini muscle originates from the ventral end of the process and courses medially to the midline to merge with its partner from the other side (Fig. 8). In adults, the tensors remain as muscle tissue within the soft palate and course inferiorly as the plug develops (Fig. 8). As a result, these muscles can facilitate elevating the plug. The levator veli palatini muscles in the fetus originate from the base of the skull at the roots of the pterygoid process and from a site related to the rostral end of the large tympanic bulla. In the adult, likely because of changes in the morphometrics of the skull during growth, particularly of the tympanic region, the levator muscles originate from the rostral side of the pterygoid process where the process attaches to the cranial base. From this far forward attachment, they are in an ideal position to act as major elevators of the oral plug.

The other two major extrinsic muscles of the palate have less or no direct relationship to the pterygoid process. The palatoglossus muscles course ventrally from the lateral cranial end of the soft palate into the tongue, and the palatoglossal folds appear joined across the midline by the caudal end of the oral part of the tongue creating a continuous U-shaped mucosal fold bracketing the plug when in the relaxed position (see Fig. 6A). The muscles are not well developed or prominent in the palatoglossal folds, most likely because their major function of closing the oropharyngeal isthmus is now performed by the oral plug. Also not well developed is the palatopharyngeus muscles that underly the narrow palatopharyngeal folds that extend onto the pharyngeal wall. Like the palatoglossus muscles, these muscles also no longer function to facilitate closing the oropharyngeal isthmus, and we observed no palatopharyngeal sphincter in the fin whale. The absence of this sphincter together with the lack of a thickened rim or flange around the upper end of the epiglottis, and our observations of the postmortem laryngeal position in the fetuses and adults, indicates to us that the upper end of the larynx is not locked into the nasopharynx as it is in odontocetes.

Two additional anatomical features are significant relative to the function of the soft palate. The first is that the intrinsic muscles in the thin caudal two-thirds of the soft palate are arranged in two layers, one oriented transversely and the other longitudinally in the dorsal plane and could dynamically change the dimensions of the soft palate. The second is that middle and inferior constrictor muscles of the pharyngeal wall attached to the hyoid apparatus and larynx are large and, together with prominent supra and infrahyoid muscles, could significantly alter the position of the larynx relative to the soft palate.

### Biomechanical model for breathing and feeding

Based on the observed anatomy, we present the following model for how the soft palate and associated anatomy is positioned during different functional states in fin whales.

**Fig. 9:**
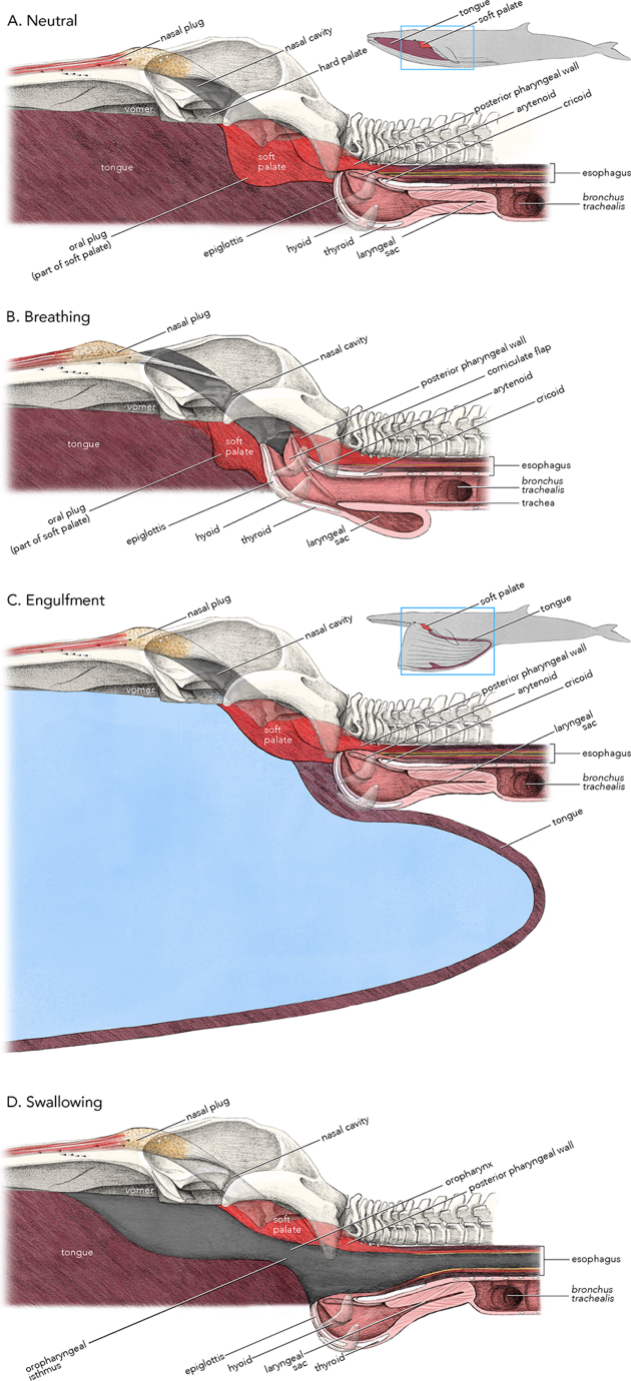
The soft palate and related structures in the neutral or rest position (**A**), and when breathing (**B**), engulfing (**C**), and swallowing (**D**). See text for details. In the figure, only one of the two nasal cavities is illustrated. The oral plug (labelled in the upper two panels) is the modified cranial part of the soft palate. See text for details. Artwork © 2024 Alex Boersma (used with permission).

In the neutral position (Fig. 9A), when the animal is not breathing or feeding, the oral plug is relaxed and fills the oropharyngeal isthmus, and the closed cranial end of the larynx is positioned at or slightly above the caudal free edge of the soft palate. This is the position we observed in the MRI scan of the fetus and in adults and would be the “rest” or “low energy” position of the structures.

During breathing (Fig. 9B), the cranial end of the larynx is positioned above the caudal end of the soft palate and the laryngeal inlet and cavity of the larynx are maximally open. Within the larynx itself, the sound-producing tissue folds are parallel to the long axis of the larynx and associated with the ventral airway wall at the entrance to the laryngeal sac (Elemans et al. 2024); hence, when the laryngeal cavity is maximally open for breathing, there is nothing protruding into the laryngeal cavity to impede the explosive respiratory airflow. Although speculative, it is also possible that the larynx moves rostrally to position the laryngeal inlet closer to the choanae and facilitate opening the nasopharynx. The soft palate may shorten in length to accommodate the forward movement of the larynx. This suggested rostral movement of the larynx together with a shortening of the soft palate is consistent with the presence of prominent muscles of the pharyngeal wall that connect with the hyoid apparatus and larynx, with the well-developed suprahyoid muscles, and with the arrangement of intrinsic muscles of the soft palate. Large translocations of the larynx have been demonstrated in some mammals (Fitch and Reby 2001; Frey et al. 2011, 2020), particularly during vocalizing, and may occur in others based on hyoid anatomy (Weissengruber et al. 2002). In adult humans, the larynx is normally in a descended position in the neck, well below the soft palate, and translocates anteriorly and upward during swallowing to facilitate closing the laryngeal inlet and opening the esophagus.

During engulfment in the fin whale (Fig. 9C), the tongue inverts and expands caudally and ventrally. Because the oropharyngeal isthmus is positioned at the top of the oral cavity, tension on the U-shaped fold (formed by the palatoglossal folds and back of tongue) that borders the plug forces the fold against the oral plug and mechanically facilitates sealing the oropharyngeal isthmus; hence, muscle activity is not needed to maintain closure of the isthmus during engulfment.

During swallowing (Fig. 9D), after prey have been concentrated and the oral cavity contracted back to a smaller volume, the levator and tensor muscles contract and elevate the oral plug to open the oropharyngeal isthmus. Contraction of muscles associated with the ventral grooved blubber, together with the retraction of the tongue into the oral cavity, increases pressure in the oral cavity that moves the slurry of krill into the pharynx. Because the cranial end of the larynx is not fixed into a nasopharyngeal position, it may be withdrawn or be moved ventrally during swallowing to maximally open the oropharynx. It is also possible that the larynx may move forward. The shape of the upper end of the epiglottis nicely cups the upper ends of arytenoids when the laryngeal inlet is closed, allowing a continuous stream of krill to flow over the larynx when positioned ventral to the soft palate. As the pharyngeal wall contracts, a continuous stream of slurry is forced through the esophagus and followed by a final peristaltic wave that clears the esophagus at the end of the swallow (Gil et al. 2024). When feeding at depth, the laryngeal sac is likely collapsed into the laryngeal cavity, further protecting the airway. An alternative hypothesis to dynamic changes in laryngeal position during swallowing is that, similar to the situation in odontocetes, the larynx remains in position with the cranial end above the level of the soft palate and that food moves through channels (piriform recesses) lateral to the large body of the larynx. Based on our observations of the anatomy of the larynx, the lack of a palatopharyngeal sphincter, and the volume of material that must move into and through the pharynx, we favor the idea that the anatomy is more dynamic and that the position of the larynx can shift during swallowing.

## Summary

In addition to the cranial end of the soft palate enlarging into an oral plug that occludes the oropharyngeal isthmus except during swallowing, a summary of the anatomical novelties acquired on a more detailed level includes: (1) the enlargement of the pterygoid processes into structurally substantial pillars that mechanically support the oral plug and provide attachment for the thickened muscular walls of the pharynx; (2) loss of the vertical belly of the tensor veli palatini muscles and the attachment of the horizontal parts directly to the ventral end of the pterygoid process; (3) “drooping” of the tensor veli palatini muscles into the oral plug resulting in the ability of the muscles to assist in elevation of the oral plug; (4) the “far forward” attachment of the levator veli palatini muscles that restricts their function to elevation of the oral plug and not to elevation of the palate as a whole; (5) the presence of longitudinal and transverse intrinsic muscle layers that presumably can alter the dimensions and shape of the soft palate caudal to the oral plug; and (6) small palatoglossal and palatopharyngeal muscles reflecting their reduced or lack of participation in closing the oropharyngeal isthmus, a function now performed by the oral plug.

In the rorqual whales, the development of the cranial end of the soft palate into an oral plug that occludes the oropharyngeal isthmus in the neutral, low energy or “rest” position and during the engulfment phase of a lunge, is one of a constellation of morphological features associated with lunge feeding and the acquisition of enormous body size in these animals.
